# Prevalence and risk factors of post-acute sequelae of SARS-CoV-2 (PASC) among veterans in the airborne hazards and open burn pit registry: a prospective, observational, nested study

**DOI:** 10.1186/s12879-024-09730-1

**Published:** 2024-08-21

**Authors:** Sherilynn M. Phen, Nisha Jani, Jacquelyn C. Klein-Adams, Duncan S. Ndirangu, Azizur Rahman, Michael J. Falvo

**Affiliations:** 1 Airborne Hazards and Burn Pits Center of Excellence, VA New Jersey Health Care System, East Orange, New Jersey USA; 2 School of Public Health, Rutgers University, Piscataway, New Jersey USA; 3 New Jersey Medical School, Rutgers University, Newark, New Jersey USA

**Keywords:** Post-acute sequelae of SARS-CoV-2 (PASC), Post-acute COVID-19 syndrome, post-COVID conditions, COVID-19, Veterans health, Open waste burning, Environmental exposure; veterans health

## Abstract

**Background:**

Veterans have unique military risk factors and exposures during deployment that may augment their risk of post-acute sequelae of SARS-CoV-2 (PASC). The purpose of this study is to identify potential risk factors for PASC among Veterans in the national Airborne Hazards and Open Burn Pit Registry (AHOBPR).

**Methods:**

This prospective observational study consisted of a semi-structured interview conducted via phone or videoconference from November 2021 to December 2022 among a stratified random sample of deployed Veterans nested within the national AHOBPR with laboratory-confirmed SARS-CoV-2 infection. PASC was defined as persistent new-onset symptoms lasting more than 2 months after initial SARS-CoV-2 infection. Deployment history, airborne hazards exposure and symptoms were obtained from the AHOBPR self-assessment questionnaire completed prior to SARS-CoV-2 infection (past). Post-infection symptoms and health behaviors obtained at study interview (present) were used to test the hypothesis that deployment experience and exposure increases the risk for PASC.

**Results:**

From a sample of 212 Veterans, 149 (70%) met criteria for PASC with a mean age of 47 ± 8.7 years; 73 (49%) were women and 76 (51%) were men, and 129 (82.6%) continued to experience persistent symptoms of SARS-CoV-2 (596.8 ± 160.4 days since initial infection). Neither exposure to airborne hazards (OR 0.97, CI 0.92–1.03) or to burn pits (OR 1.00, CI 0.99-1.00) augmented risk for PASC.

**Conclusions:**

PASC is highly common among Veterans enrolled in the AHOBPR, but we did not observe any unique military risk factors (e.g., airborne hazards exposure) that augmented the risk of PASC. Our findings may provide guidance to clinicians in the VHA network to administer appropriate care for Veterans experiencing PASC.

**Supplementary Information:**

The online version contains supplementary material available at 10.1186/s12879-024-09730-1.

## Background

Over 9 million veterans receive care through the Veterans Health Administration (VHA), the largest integrated health care system in the United States, of which more than 900,000 veterans have been tested or treated for known or probable severe acute respiratory syndrome coronavirus 2 (SARS-CoV-2) infection [1]. Among veterans infected with SARS-CoV-2, also referred to as COVID-19, approximately 13.5–45% [2, 3] experience residual symptoms such as fatigue, shortness of breath, and cognitive dysfunction that persist for several months following their initial infection and are referred to as post-acute sequelae SARS-CoV-2 infection (PASC). Despite variability in PASC prevalence, there is consistency with respect to risk factors – i.e., older age, more severe initial clinical presentation, and greater comorbidity burden [4–6]. Moreover, PASC burden is substantial in veterans and appears greatest among those with poorer baseline health [7]. As veterans are known to have poorer health and health behaviors relative to civilians [8], particularly those who utilize the Department of Veterans Affairs (VA) for their health care [9], this vulnerable population is deserving of attention.

During military service, veterans are exposed to a variety of environmental hazards that may contribute to specific health problems following their deployment. For example, veterans of recent conflicts in Iraq, Afghanistan, and surrounding regions were deployed to regions characterized by fine particulate matter levels that far exceeded military and domestic exposure guidelines [10, 11]. Elevated particulate matter levels in the deployment environment reflect regional conditions (e.g., sand and dust) as well as anthropogenic activities (e.g., smoke from open burn pits and industrial air pollution) that may predispose veterans to adverse health outcomes. Whether these same deployment exposures increase the risk of COVID-19 as well as PASC among veterans has not been thoroughly investigated, despite strong biological plausibility [12].

To investigate the prevalence and potential risk factors of PASC among Veterans with deployment-related environmental exposures, we conducted a prospective observational study nested in the national Airborne Hazards and Open Burn Pit Registry (AHOBPR). More than 500,000 service members and veterans are currently enrolled in the AHOBPR, which was launched in 2014 to understand the potential health effects of exposure to airborne hazards and burn pits during military services. Through the AHOBPR, participants reported their exposure, symptoms, and state of health prior to becoming infected by SARS-CoV-2, thereby providing a baseline for comparing their experience with COVID-19 and present health status. We leveraged this type of natural experiment to understand the prevalence of PASC in this vulnerable population and to identify potential factors that may augment risk. These preliminary data may help guide the VHA and clinicians in caring for veterans who are struggling with PASC.

## Methods

### Study design and participants

Veterans with COVID-19 were identified from the Veterans Health Administration (VHA) electronic health record who had a polymerase chain reaction (PCR) test positive for SARS-CoV-2 between March 15, 2020, and October 7, 2021, were identified from the VHA electronic health record. Those with positive PCR diagnoses were cross-referenced with AHOBPR participants to identify the study sample population. The AHOBPR is a volunteer environmental registry with enrollment limited to servicemembers and veterans who were deployed to one or more locations in the Southwest Asia theater of operations at any time after August 2, 1990, or Afghanistan or Djibouti after September 11, 2001 [13]. Those who requested not to be contacted and those with VHA electronic health record diagnoses of major psychotic disorder or dementia were excluded. The remaining sample was divided into strata based on sex, race, and the year of COVID-19 diagnosis as covariates. Simple random sampling was used to create blocks of ≤ 48 individuals, who were subsequently mailed study invitation letters, screened, and scheduled by phone in a staggered fashion. This nested, prospective, observational study within the AHOBPR was submitted to the Veterans Affairs New Jersey Institutional Review Board and determined exempt from human subjects research (Category 2 and 3). This study was therefore under the purview and oversight of the Veterans Affairs New Jersey Research and Development Committee (#1584926). Verbal consent was obtained from all participants and a waiver of documentation of consent was granted. All procedures conformed to the Declaration of Helsinki and reporting of results followed the STROBE guidelines [14].

### Procedures

Veterans who were eligible and interested in the study provided their verbal consent to participate in a telephone or video-based (VA Virtual Video Connect) interview. Each interview lasted approximately 60 min and was conducted by a single investigator for all study participants from November 2021 to December 2022. Veterans’ responses were collected and entered in real-time using the web-based software application Research Electronic Data Capture (REDCap), hosted by the Department of Veterans Affairs (VA) on its intranet [15, 16]. The interviews comprised a series of standardized and modified surveys designed to comprehensively characterize each veteran’s COVID-19 experience, symptoms at present and at the time of infection, medical history including self-reported comorbidities at time of interview, and lifestyle. We used the COVID-19 Questionnaire (version 1.0) designed by the SubPopulations and InteRmediate Outcome Measures In COPD Study (SPIROMICS), which was funded by the Collaborative Cohort of Cohorts for COVID-19 Research [17]. The COVID-19 Questionnaire included a modified version of the FLU-PRO^®^ [18] questionnaire (Supplementary Appendix, Table S1). Additionally, each participant completed the CDC’s Chronic Fatigue Syndrome Symptom Inventory [19], Multi-Dimensional Fatigue Inventory [20], Dyspnea-12 questionnaire [21] and the modified Medical Research Council dyspnea scale.

Prior to becoming infected by SARS-CoV-2, study participants completed the AHOBPR’s self-assessment questionnaire, which included detailed information about each veteran’s deployment as well as their self-reported exposures during deployment. Except blast overpressure, veterans who endorsed exposure (yes/no) also estimated their average exposure to airborne hazards in days over a typical month. Airborne hazard exposure was operationalized to include convoy operations, pesticides, engine maintenance, heavy smoke, refueling operations, and construction. Burn pit exposure was assessed separately, whereby participants reported the average number of hours of burn pit smoke exposure per day for each deployment segment, which was multiplied by the number of days for that deployment. These values were summed for the cumulative hours of burn pit smoke exposure for all deployments. Veterans also rated their health status at the time of questionnaire completion, as well as their concerns for future health problems. Additional details and item-level questions are provided in the Supplementary Appendix. The AHOBPR-derived data collected prior to their COVID-19 diagnosis were merged with the data obtained during their interviews.

### Statistical analysis

We used a modified clinical case definition of PASC [22] which we defined as (1) PCR-based diagnosis of COVID-19, (2) confirmation of COVID-19 by self-report, (3) and new-onset symptoms (or symptom exacerbation) that persisted for ≥ 60 days post-infection. Veterans meeting this case definition were classified as ‘PASC’ and those who did not were considered ‘Recovered’.

Descriptive statistical analysis was performed for all variables by stratifying cases and controls. Bivariate analyses and chi-square tests (χ^2^) were performed for categorical variables and t-tests were performed for continuous variables to assess differences. Multivariable logistic regression of cases and controls, demographic factors (age, sex, and BMI), COVID-19 variant (ancestry, Alpha, and Delta), and recorded comorbidities (cardiac, neurological, gastrointestinal, and respiratory) for associations between case status (yes vs. no) were performed. We reported the odds ratios and 95% confidence intervals for each variable included in the model. This initial model was extended to consider military deployment history and experience, including exposure to airborne hazards. Using the same methods, an exploratory analysis was performed for associations between exposures and hospitalizations and length of hospital stay.

Differences between PASC and Recovered were tested with a two-sided χ^2^ test for categorical variables or a one-sided *t* test (or Wilcoxon signed rank test for non-normally distributed variables) for continuous variables. Standardized effect calculations, with Hedges’ *g* correction, were computed along with 95% confidence intervals [23]. Effect sizes were interpreted as small (*g* = 0.2), moderate (*g* = 0.5), or large (*g =* 0.8).

## Results

**Fig. 1:**
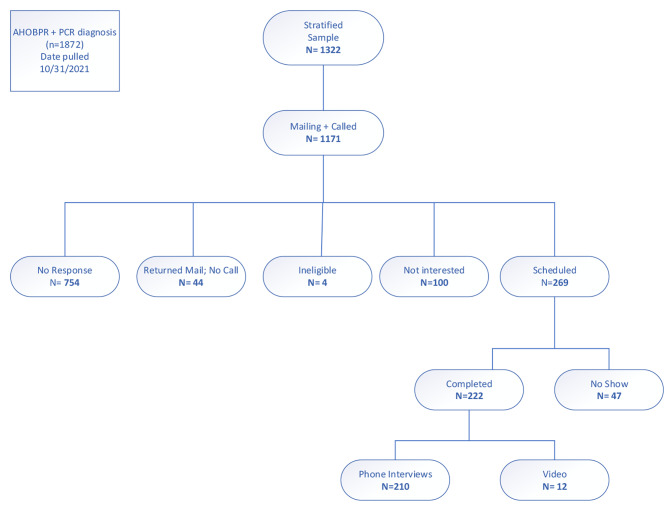
Veteran participants in the airborne hazards and open burn pit registry (AHOBPR) with a positive polymerase chain reaction (PCR) were identified and recruited to participate in the study

As of October 2021, 7,178 veterans enrolled in the AHOBPR had a PCR diagnosis of COVID-19 within the VHA electronic health record. As illustrated in Figs. 1 and 322 of these veterans were assigned to our three strata (sex, race, and diagnosis year), and 222 completed the study (Fig. 1). Ten Veterans were excluded from the final analyses (9 were ≤ 60 days since the last COVID infection; 1 did not recall having a PCR test within the VHA). Of the remaining 212 veterans, 70% (*n* = 149) met the criteria for PASC, with the remainder recovering. Among those with PASC, the majority (123/149, 82.6%) continued to experience persistent post-COVID symptoms (≥ 90–982 days) at the time of study participation. The remaining individuals (*n* = 26) reported recovery after study participation (≥ 90–730 days).

Participant demographics and characteristics are reported in Table 1 separately for the total analyzed study sample (*n* = 212), Veterans with PASC (*n* = 149), and those who recovered (*n* = 63). Demographics (Table 1) and military experience (Table 2) were similar between Veterans with PASC and recovery, except age and body mass index, whereby Veterans with PASC were younger and more obese. In addition, Veterans with PASC self-reported more respiratory comorbidities at the time of questionnaire completion. The demographics of our nested sample were also compared to the broader AHOBPR, delimited to those utilizing VHA care (Supplementary Appendix Table S2). Note that comparisons were made using data obtained at the time of AHOBPR completion, which predated the present study. The mean time between AHOBPR completion and PASC interview for the total cohort was 5.07 ± 2.28 years, the PASC group 4.98 ± 2.27 years and the recovered group 5.29 ± 2.30 years. Our study sample comprised more women veterans by design (47.2% vs. 11.1%), but also differed by having a greater proportion who served in the army (72.6% vs. 59.6%), endorsed greater functional limitations (i.e., walking, stairs, walking up a hill), and self-reported respiratory conditions (i.e., allergies, asthma, and COPD).

**Table 1:** Participants’ sociodemographics and comorbidities

	PASC	Recovered	Total Sample	*P*-Value
**N**	149	63	212	
**Age**,** years**	47.06 ± 8.6	49.44 ± 9.7	47.76 ± 9.0	0.09
**BMI n (%)**				< 0.01
Underweight (< 18.5)	0 (0.0)	0 (0.0)	0	
Normal (18.5–24.9)	14 (9.4)	13 (20.5)	27	
Overweight (25.0-29.9)	40 (26.8)	23 (36.5)	63	
Obese (30.0+)	95 (63.8)	27 (42.9)	122	
**Sex**				0.69
Male	76 (51.0)	34 (53.9)	110	
Female	73 (49.0)	29 (46.0)	102	
**Race**				0.70
Black	63 (42.3)	25 (39.7)	88	
White	57 (38.3)	31 (49.2)	88	
Other^†^	29 (19.4)	7 (11.1)	36	
**Ethnicity**				0.65
Hispanic	25 (16.8)	9 (14.3)	34	
Non-Hispanic	124 (83.2)	54 (85.7	178	
**Education Level**				0.09
High school	4 (2.7%)	6 (9.5)	10	
Some college/ Technical Schools	46 (30.9%)	18 (28.6)	64	
College Graduate	99 (66.4%)	39 (61.9)	138	
**Income Level**				0.96
15,000–50,000	18 (12.1)	9 (14.3)	27	
50,000–75,000	35 (23.5)	14 (22.2)	49	
> 75,000	74 (49.7)	30 (47.6)	104	
Missing^‡^	22 (14.8)	10 (15.9)	32	
**Urbanicity**				0.86
Urban	31 (21.8)	15 (23.8)	46	
Suburban	82 (55)	33 (52.4)	115	
Rural	33 (22.1)	13 (20.6)	46	
Don’t know	3 (2.0)	2 (3.2)	5	
**Smoking History**				0.77
Current smoker	10 (6.7)	6 (9.5)	16	
Former smoker	62 (41.6)	25 (39.6)	87	
Never smoked	77 (51.6)	32 (50.7)	109	
**Comorbidities**				
Respiratory				0.04
*Yes*	119 (79.8)	42 (66.6)	161	
*No*	30 (20.1)	21 (33.3)	51	
Cardiovascular				0.69
*Yes*	100 (67.1)	44 (69.8)	144	
*No*	49 (32.9)	19 (30.1)	68	
Gastrointestinal				0.26
*Yes*	113 (75.8)	43 (68.3)	156	
*No*	36 (24.1)	20 (31.2)	56	
Neurological				0.48
*Yes*	137 (91.9)	56 (88.9)	193	
*No*	12 (8.0)	7 (11.1)	19	

Note: Information in this table was acquired at time of study interview. †Other race includes Native American, Pacific Islander, more than one race and any other race as self-reported during interview. ‡Missing responses under income level indicate those not knowing their income level or not wishing to disclose their income

**Table 2:** Participants’ military history, deployment experience and airborne hazards exposure

	PASC	Recovered	Total Sample	*P*-Value
**N**	149	63	212	-
**Military Branch**				0.25
Air Force	21 (14.1)	8 (12.7)	29	
Army	104 (69.8)	50 (79.4)	154	
Marine	10 (6.7)	2 (3.2)	12	
Navy	14 (9.4)	2 (3.2)	16	
Coast Guard	-	1 (1.6)	1	
**Classification of Service**				0.40
National Guard	16 (10.7)	6 (9.5)	22	
Regular	110 (73.8)	51 (80.9)	161	
Reserves	23 (15.4)	6 (9.5)	29	
**Conflicts Served**				**-**
Bosnia/ Croatia/ Kosovo	6 (4.0)	4 (6.3)	10	
Persian Gulf War	10 (6.7)	7 (11.1)	17	
Operation Enduring Freedom	79 (53.0)	26 (41.3)	105	
Operation Iraqi Freedom	105 (70.5)	44 (69.8)	149	
Operation New Dawn	6 (4.0)	3 (4.8)	9	
Operation Provide Comfort	0 (0)	2 (3.2)	2	
Operation Southern Watch	3 (2.0)	0 (0)	3	
Operation Desert Fox	1 (0.7)	2 (3.2)	3	
Operation Freedom Sentinel	1 (0.7)	1 (1.6)	2	
Somalia	2 (1.3)	1 (1.6)	3	
**Cumulative deployment length (days)**	412.2 ± 282.2	406.0 ± 264.6	410.4 ± 276.5	0.89
**Airborne hazards exposure (days·month** ^**− 1**^ **)†**	7.8 ± 6.1	6.8 ± 7.2	7.51 ± 6.5	0.34
**Burn pit exposure (days)‡**	312 ± 267.9	296.3 ± 244.7	308.0 ± 260.8	0.66

†Airborne hazards exposure was average exposure to convoy operations, pesticides, engine maintenance, heavy smoke, refueling operations, and construction in days over a typical month of deployment. ‡ Burn pit exposure was total cumulative exposure in days

COVID-19 diagnoses in the VHA medical record occurred between March 2020 and August 2021, with the majority of our sample being diagnosed when the ancestral strain was most common in circulation (74.0%). Overall, Veterans participated in this study an average of 589.4 days after their initial diagnosis (Table 3). At the time of diagnosis, 12.1% of Veterans with PASC were fully vaccinated compared with 22.2% of veterans who recovered (χ^2^ = 3.55, *p* = 0.059; Table 3). Approximately 11.2% were hospitalized at the time of diagnosis and those with PASC were more likely (PASC vs. recovered: 14.1% vs. 6.35%, (χ^2^ = 2.55, *p* = 0.110; Table 3), but no veterans in our sample were admitted to intensive care. Additional details regarding the veterans’ COVID-19 experience are reported in Table 3.

**Table 3:** COVID-19 temporal characteristics, experience and severity among veterans with PASC and those who recovered

	PASC	Recovered	Total Sample	*P*-Value
**N**	149	63	212	-
**Predominant Variant**				0.02
Ancestral	115 (77.2)	42 (66.7)	157	
Alpha	20 (13.4)	14 (22.2)	34	
Delta	14 (9.4)	7 (11.1)	21	
**Vaccination Status at Diagnosis** ^†^				0.06
Unvaccinated	131 (87.9)	49 (77.8)	180	
Fully Vaccinated	18 (12.1)	14 (22.2)	32	
**Date of Diagnosis to time of interview (days)**	596.83 ± 160.36	571.86 ± 165.96	589.4 ± 162.05	0.34
**Number of times tested**	8.17 ± 9.40	6.57 ± 9.20	7.7 ± 9.3	0.03
**Number of positive COVID tests**	1.37 ± 0.661	1.24 ± 0.429	1.3 ± 0.6	0.34
**Covid Severity**				
Hospitalization (days)				0.11
0	128 (85.9)	59 (93.7)	187 (88.2)	
1–7	9 (6.0)	4 (6.3)	13 (6.1)	
8–14	8 (5.4)	0	8 (3.8)	
15–21	3 (2.0)	0	3 (1.4)	
> 21	1 (0.7)	0	1 (0.5)	
Total	1.54 ± 5.55	0.14 ± 0.64	1.12 ± 4.71	< 0.01
**Symptoms at time of COVID infection†**				
Fever	110 (73.8)	31 (49.2)	141 (66.5)	< 0.01
Trouble breathing	116 (77.9)	29 (46.0)	145 (68.4)	< 0.01
Chest congestion	102 (68.5)	25 (39.7)	127 (59.9)	< 0.01
Chest tightness	95 (63.8)	20 (31.7)	115 (54.2)	< 0.01
Dry or hacking cough	107 (71.8)	32 (50.8)	139 (65.6)	< 0.01
Wet or loose cough	78 (52.3)	13 (20.6)	91 (42.9)	< 0.01
Body aches or pains	134 (89.9)	37 (58.7)	171 (80.7)	< 0.01
Chills or shivering	110 (73.8)	33 (52.4)	143 (67.5)	< 0.01
Sore or painful throat	92 (61.7)	21 (33.3)	113 (53.3)	< 0.01
Congested or stuffy nose	99 (66.9)	28 (44.4)	127 (59.9)	< 0.01
Runny or dripping nose	65 (43.6)	17 (27.0)	82 (38.7)	0.02
Diarrhea	69 (46.3)	13 (20.6)	82 (38.7)	< 0.01
Weak or tired	143 (96.0)	43 (68.3)	186 (87.7)	< 0.01
Loss of smell	103 (69.1)	26 (41.3)	129 (60.8)	< 0.01
Loss of taste	104 (69.8)	27 (42.9)	131 (66.2)	< 0.01
Overall Score	0.68 ± 0.22	0.54 ± 0.23	0.65 ± 0.23	< 0.01
**Overall symptom severity**				< 0.01
None	-	14 (22.2)	14 (6.6)	
Mild	9 (6)	9 (18.4)	18 (8.5)	
Moderate	29 (19.5)	18 (36.7)	47 (22.2)	
Severe	67 (45)	18 (36.7)	85 (40.1)	
Very Severe	44 (29.5)	4 (8.2)	48 (22.6)	
**Impact of symptom on daily life**				< 0.01
Not at all	3 (2.0)	4 (8.2)	7 (3.5)	
A little bit	2 (1.3)	2 (4.1)	4 (2.0)	
Somewhat	9 (6.0)	10 (20.4)	19 (9.6)	
Quite a bit	13 (8.7)	8 (16.3)	21 (10.6)	
Very much	122 (81.9)	25 (51.0)	147 (74.2)	

†Symptoms were assessed using the modified FLU-PRO Plus survey

Adjusted logistic regression analysis (Table 4) demonstrated no change in the odds of PASC with increasing age (OR 1.02, CI 0.99–1.06) or BMI (0.92, 0.86–0.98). Neither airborne hazard exposure (OR 0.97, CI 0.92–1.03) nor burn pit exposure (OR 1.00, CI 0.99-1.00) contributed to a change in risk for PASC.

**Table 4:** Multivariable logistic regression models to predict PASC based upon sociodemographic factors and comorbidities (model 1) and deployment factors (model 2)

	Model 1		Model 2	
	OR	95% CI	OR	95% CI
**Age (years)**	1.02	0.99, 1.06	1.02	0.99, 1.06
**BMI (kg/m** ^**2**^ **)**	0.92	0.86, 0.98	0.92	0.86, 0.98
**Sex**				
Male	-	-	-	-
Female	0.81	0.42, 1.58	0.75	0.38, 1.51
**Race/Ethnicity**				
White	-	-	-	-
Black	0.98	0.47, 2.02	0.99	0.48, 2.06
Other	0.77	0.17, 3.46	0.78	0.17, 3.55
Missing	0.94	0.35, 2.49	1	0.37, 2.72
**Variant**				
Ancestry	-	-	-	-
Alpha	1.84	0.78, 4.32	1.80	0.76, 4.27
Delta	1.46	0.51, 4.14	1.46	0.51, 4.15
**Respiratory**				
No	-	-	-	-
Yes	0.55	0.27, 1.12	0.55	0.26, 1.13
**Cardiovascular**				
No	-	-	-	-
Yes	1.27	0.61, 2.63	1.34	0.63, 2.84
**Gastrointestinal**				
No	-	-	-	-
Yes	0.83	0.39, 1.77	0.82	0.38, 1.75
**Neurological**				
No	-	-	-	-
Yes	0.72	0.24, 2.13	0.75	0.25, 2.26
**Cumulative deployment length (days)**	-	-	1	0.99, 1.00
**Airborne hazards exposure (days·month** ^**− 1**^ **)†**	-	-	0.97	0.92, 1.03
**Burn pit exposure (days)‡**	-	-	1	0.99, 1.00

†Airborne hazards exposure was average exposure to convoy operations, pesticides, engine maintenance, heavy smoke, refueling operations, and construction in days over a typical month of deployment. ‡ Burn pit exposure was total cumulative exposure in days

Additionally, airborne hazard exposure (OR 1.00, CI 0.99-1.00) nor burn pit exposure (OR 0.99, CI 0.99-1.00) were associated with odds of hospitalization and length of hospitalization (results not shown).

The veterans’ current health status and symptoms at the time of their interviews are reported in Table 5. On an average, our sample did not engage in the recommended minimum physical activity and endorsed a considerable symptom burden. As expected, Veterans with PASC endorsed greater overall symptoms than those who had recovered, with the largest effect (*g* = 0.62 [0.32, 0.92]) demonstrated for dyspnea on the modified Medical Research Council dyspnea scale. The detailed results are listed in Table 5.

**Table 5:** Current symptoms of fatigue and dyspnea and self-reported physical activity levels assessed during the time of study interview

	PASC	Recovered	Total Sample	*P*-Value	Effect Size (95%CI)
**N**	149	63	212	-	-
**Multidimensional Fatigue Inventory**					
General Fatigue	11.8 ± 2.4	12.1 ± 2.1	11.9 ± 2.3	0.16	-0.15 (-0.44, 0.15)
Physical Fatigue	12.9 ± 1.9	13.1 ± 1.7	13.0 ± 1.86	0.35	-0.06 (-0.35, 0.24)
Vigor	12.7 ± 2.2	12.7 ± 2.3	12.7 ± 2.2	0.44	-0.02 (-0.27, 0.32)
Reduced Motivation	11.3 ± 2.6	12.1 ± 2.6	11.6 ± 2.6	0.04	-0.26 (-0.58, 0.03)
Mental Fatigue	11.5 ± 1.8	11.7 ± 1.5	11.5 ± 1.7	0.19	-0.13 (-0.42, 0.17)
**Dyspnea-12 Questionnaire**					
Total Score	8.0 ± 8.2	5.9 ± 6.9	7.4 ± 7.9	0.03	0.29 (-0.01, 0.58)
Physical Component	5.4 ± 4.8	4.1 ± 4.3	5.0 ± 4.7	0.03	0.28 (-0.01, 0.58)
Emotional Component	2.7 ± 3.8	1.8 ± 3.0	2.4 ± 3.6	0.05	0.25 (-0.05, 0.55)
**Modified Medical Research Council Dyspnea Scale (0–4)**	1.3 ± 1.0	0.7 ± 0.8	2.1 ± 1.0	< 0.001	0.62 (0.32, 0.92)
**CDC Chronic Fatigue Symptom Inventory (Total Score)**	78.3 ± 47.4	56.8 ± 38.6	72.1 ± 46.0	0.001	0.48 (0.12, 0.78)
**Physical Activity (min·wk** ^**− 1**^ **)**					
Vigorous	20.6 ± 33.8	23.3 ± 29.8	21.4 ± 32.6	0.29	-0.08 (-0.38, 0.21)
Moderate	30.7 ± 43.1	35.2 ± 40.5	32.0 ± 42.3	0.24	-0.11 (-0.40, 0.19)
Walking	37.0 ± 54.5	35.2 ± 32.0	36.5 ± 48.8	0.39	0.04 (-0.26, 0.33)

## Discussion

In this prospective, observational study nested within the Airborne Hazards and Open Burn Pit Registry (AHOBPR), we found that 70% of our sample with a PCR diagnosis of COVID-19 met the clinical case definition of post-acute sequelae of SARS-CoV-2 (PASC), whereby chronic dyspnea appears to be the most burdensome and persistent symptom. However, we did not identify any unique military risk factors (e.g., airborne hazard exposure, Supplementary Appendix, Table S3) in our sample that enhanced the risk of PASC.

Ioannu and colleagues [2] recently highlighted the considerable variability in the proportion of veterans receiving ongoing care and management secondary to acute SARS-CoV-2 infection. Using diagnostic codes for COVID-19, investigators observed that the proportion of veterans receiving long-term COVID care ranged from 3.0 to 41.0% across 171 medical centers. Such information (e.g., proportion receiving care, regional variability) is essential for large healthcare systems to plan effectively, including identifying risk factors for those veterans requiring persistent long-COVID care. Investigators found that older veterans who were unvaccinated and had significant comorbidities were at the greatest risk for PASC [2]. Despite the strengths of this large cohort study; unique factors related to military service (i.e., environmental exposures) were not considered. Attention to military exposures and their impact on post-deployment health is increasingly recognized particularly after passage of the Sergeant First Class Heath Robinson Honoring Our Promise to Address Comprehensive Toxins (PACT) Act [24, 25]. The PACT Act considerably expands and extends eligibility for health care to Veterans with military environmental exposures from the Vietnam, Gulf War and post-9/11 eras; therefore, planning for the unique needs of these Veterans in concert with PASC or long-COVID care is essential.

Prior to infection at the time of AHOBPR questionnaire completion, Veterans with PASC were younger, more obese and endorsed more respiratory co-morbidities (Table 1). Their military experience and exposures, however, were similar to those without PASC which did not support our hypothesis. There are several potential explanations for these findings, most notably the small sample size of this preliminary study, as well as the lack of objective individual- or area-level exposure data. Reliance upon self-reported exposures (e.g., recall bias) is a notable limitation of research on post-deployment respiratory health [26]; however, significant efforts are underway to derive semi-quantitative measures of particulate matter exposure [27] that could be explored in future studies. In between-group analyses, it appears that Veterans with PASC were less likely to be fully vaccinated at the time of infection (PASC vs. recovered; 12.1% vs. 22.2%), which is consistent with the findings observed across the entire VHA [2]. However, vaccination status as well as other factors that differed between groups (i.e., age, BMI, respiratory co-morbidities) did not augment the risk of PASC in our full model.

Veterans with PASC endorsed greater symptom burden at the time of infection (Table 3) that persisted throughout the time of their study interview (Table 5) in comparison to those who recovered. The latter reflects the clinical case definition used to identify Veterans with PASC, but more importantly highlights that dyspnea appears to be the most prominent post-COVID symptom as it demonstrated the largest effect size between groups (*g* = 0.62; Table 5). Prior to becoming infected by COVID and at the time of AHOBPR questionnaire completion, veterans who went on to develop PASC reported greater respiratory comorbidities (80% vs. 67%; Table 3) – specifically, asthma and COPD (Supplementary Appendix, Table S4). Interestingly, dyspnea severity as assessed by the modified Medical Research Council dyspnea scale was similar between groups at the time of questionnaire completion (pre-COVID). At the time of their study interview, veterans with PASC reported a 41% increase in dyspnea severity relative to their pre-COVID levels whereas those who recovered had no increase in dyspnea (Supplementary Appendix, Figure S1). These findings suggest that those with self-reported obstructive lung disease may be an important sub-population within the AHOBPR for focused medical surveillance.

Notwithstanding the limitations of an environmental registry [28], our sample within the national AHOBPR has several design strengths. A key strength was access to a national sample of veterans (United States and its territories) who had completed the AHOBPR, accessed VHA for care, and had a verified COVID-19 diagnosis in the electronic health record. From this large sample, we were able to implement a probability sampling approach that could ensure adequate representation of the sex, race, and timing of COVID-19 diagnosis. This approach reduces sampling bias and improves the accuracy of our results. For example, by stratifying by year of COVID-19 diagnosis, we were able to partly control the predominant SARS-CoV-2 variant in circulation as well as account for availability of vaccines at time that could attenuate the risk of PASC [29]. Stratifying by sex was important to account for overall increased risk of PASC [30] and female sex-specific health risks of SARS-CoV-2 infection [31, 32], but also to ensure female Veterans’ health concerns were not overlooked in a male dominated population [33]. Additionally, we stratified by race given the disproportionate burden of COVID-19 in communities of color [30, 34, 35]. Participation bias may also be a limitation of our approach as the AHOBPR is a volunteer registry and those experiencing PASC symptoms may have been more likely to participate. Overall, the present study illustrates the potential utility of the AHOBPR for hypothesis-generation research.

## Conclusion

Our study revealed that 70% of Veterans in our sample who were infected with COVID-19 exhibited PASC, with chronic respiratory difficulties appearing to be the most burdensome and persistent symptom. The findings of this nested observational study in a national registry highlight the substantial prevalence of PASC in this Veteran population. Although veterans’ deployment exposures did not contribute to their risk of PASC, their service-related health concerns along with PASC should not be disregarded when considering their overall state of health as they seek care.

### Electronic supplementary material

Below is the link to the electronic supplementary material.

Supplementary Material 1

Supplementary Material 2

## Data Availability

Metadata supporting the findings of this study are available from the corresponding author upon reasonable request. There were no provisions for making individual data records publicly available. The contents do not represent the views of the U.S. Department of Veterans Affairs or the United States Government.

## Abbreviations

VHA: Veterans Health Administration
PASC: Post-acute sequelae SARS-CoV-2 infection
VA: Veteran Affairs
AHOBPR: Airborne Hazards and Open Burn Pit Registry
REDCap: Research Electronic Data Capture
SPIROMICS: SubPopulations and InteRmediate Outcome Measures In COPD Study
