# Supplementary material for: Prevalence and risk factors of post-acute sequelae of SARS-CoV-2 (PASC) among veterans in the airborne hazards and open burn pit registry: a prospective, observational, nested study
Source: BMC Infect Dis. 2024 Aug 21;24:846. doi: 10.1186/s12879-024-09730-1 (PMC11337853; doi:10.1186/s12879-024-09730-1)
Supplement: Supplementary file 1 — Supplementary Material 1 [file 12879_2024_9730_MOESM1_ESM.docx]

# **SUPPLEMENTARY APPENDIX**

## Additional Questionnaire Details

Table S1. Modified FLU-PRO® Plus
The original FLU-Pro Plus contains 34 items. Our survey asked about 15 symptoms, which are listed below: If a symptom was endorsed by answering “yes” then the severity of the symptom was asked. A “no” response resulted in 0. The total score was derived using the mean score for all the symptoms.

|  | Not at all  1 | A little bit 2 | Somewhat 3 | Quite a bit 4 | Very much 5 |
| --- | --- | --- | --- | --- | --- |
| Fever |  |  |  |  |  |
| Trouble Breathing |  |  |  |  |  |
| Chest Congestion |  |  |  |  |  |
| Chest Tightness |  |  |  |  |  |
| Dry or hacking cough |  |  |  |  |  |
| Wet or loose cough |  |  |  |  |  |
| Body aches or pains |  |  |  |  |  |
| Chills or shivering |  |  |  |  |  |
| Sore or painful throat |  |  |  |  |  |
| Congested or stuffy nose |  |  |  |  |  |
| Runny or dripping nose |  |  |  |  |  |
| Diarrhea |  |  |  |  |  |
| Weak or tired |  |  |  |  |  |
| Loss of smell |  |  |  |  |  |
| Loss of taste |  |  |  |  |  |

## Airborne Hazards and Open Burn Pit Registry (AHOBPR) Data

Data were obtained from participants of the AHOBPR who were enrolled in the Veterans Health Administration (VHA) for medical care from 01/01/2014 to 10/31/2021 (n=233,116). Veterans who participated in our study (Study Sample, n = 212) were compared to AHOBPR participants who were enrolled in VHA care (AHOBPR Participants, n = 232,904) on demographics (Table S1), symptoms and medical history (Table S2), and airborne hazards exposure (Table S3). In addition, we also compared Veterans with PASC (n = 149) with those who recovered (n = 63) in our Study Sample. Categorical and continuous variables were compared using chi-square (χ^2^) and Wilcoxon-Mann-Whitney tests, respectively.

### Table S2: Cohort Demographics

| **Characteristic, N (%)** | **PASC (N=149)** | **Recovered (N=63)** | **Study Sample (N=212)** | **AHOBPR Participants (N=232,904)** | **P-value^a^** | **P-value^b^** |
| --- | --- | --- | --- | --- | --- | --- |
| **Age†** |  |  |  |  | 0.073 | 0.142 |
| 20 and below | 26 (17.4) | 11 (17.5) | 37 (17.4) | 33721 (14.5) |  |  |
| 21-40 | 60 (40.3) | 21 (33.3) | 81 (38.2) | 107985 (46.4) |  |  |
| 41-60 | 63 (42.3) | 29 (46.0) | 92 (43.4) | 87322 (37.5) |  |  |
| 60 and above | - | 2 (3.2) | 2 (0.9) | 3876 (1.7) |  |  |
| **Gender** |  |  |  |  | <0.001 | 0.605 |
| Male | 77 (51.7) | 35 (55.6) | 112 (52.8) | 206756 (88.9) |  |  |
| Female | 72 (48.3) | 28 (44.4) | 100 (47.2) | 25938 (11.1) |  |  |
| **Branch of Service** |  |  |  |  | 0.003 | 0.395 |
| Army | 107 (70.5) | 49 (77.8) | 154 (72.6) | 138741 (59.6) |  |  |
| Coast Guard | - | 1 (1.6) | 1 (0.5) | 486 (0.2) |  |  |
| Air Force | 19 (12.7) | 8 (12.7) | 27 (12.7) | 47239 (20.3) |  |  |
| Marine Corps | 11 (7.4) | 2 (3.2) | 13 (6.1) | 25850 (11.1) |  |  |
| Navy | 10 (6.7) | 2 (3.2) | 12 (5.7) | 17104 (7.3) |  |  |
| Public Health | - | - | - | 32 (0.01) |  |  |
| Unknown | 4 (2.7) | 1 (1.6) | 5 (2.4) | 3452 (1.5) |  |  |

*†Age = age at time of AHOBPR self-assessment questionnaire completion; ^a^ Comparison of study sample to those of the AHOBPR; ^b^ Comparison of PASC vs. Recovered*

### Table S3: Airborne Hazards Exposure

| **Characteristic, N (%)** | **PASC (N=149)** | **Recovered (N=63)** | **Study Sample (N=212)** | **AHOBPR Participants (N=232,904)** | **P-value^a^** | **P-value^b^** |
| --- | --- | --- | --- | --- | --- | --- |
| **Close enough to feel the blast from IED, N (%)** |  |  |  |  | 0.915 | 0.718 |
| Yes | 103 (69.1) | 41 (65.1) | 144 (67.9) | 157897 (67.8) |  |  |
| No | 40 (26.8) | 18 (28.6) | 58 (27.4) | 62556 (26.9) |  |  |
| Other/missing | 6 (4.0) | 4 (6.3) | 10 (4.7) | 12451 (5.3) |  |  |
| **Near heavy combat smoke (days·month^-1^)** |  |  |  |  |  |  |
| Yes | 101 (67.8) | 36 (57.1) | 137 (64.6) | 143710 (61.7) | 0.382 | 0.138 |
| No | 48 (32.2) | 27 (42.9) | 75 (35.4) | 89194 (38.3) |  |  |
| Mean (SD) | 12.28 (12.07) | 9.76 (12.62) | 11.53 (12.26) | 10.8 (12.12) | 0.377 | 0.142 |
| Median (Range) | 10 (0-31) | 2 (0-31) | 7 (0-31) | 5 (0-31) |  |  |
| **Convoy (days·month^-1^)** |  |  |  |  |  |  |
| Yes | 111 (74.5) | 43 (68.3) | 154 (72.6) | 171433 (73.6) | 0.750 | 0.351 |
| No | 38 (25.5) | 20 (31.7) | 58 (27.4) | 61471 (26.4) |  |  |
| Mean (SD) | 13.46 (12.01) | 10.97 (12.35) | 12.72 (12.14) | 13.68 (12.13) | 0.3229 | 0.196 |
| Median (Range) | 10 (0-31) | 5 (0-31) | 10 (0-31) | 14 (0-31) |  |  |
| **Refueling operations (days·month^-1^)** |  |  |  |  |  |  |
| Yes | 96 (64.4) | 32 (50.8) | 128 (60.3) | 148600 (63.8) | 0.299 | 0.063 |
| No | 53 (35.6) | 31 (49.2) | 84 (39.6) | 84304 (36.2) |  |  |
| Mean (SD) | 10.44 (11.61) | 8.22 (11.17) | 9.78 (11.5) | 11.11 (12) | 0.126 | 0.107 |
| Median (Range) | 5 (0-31) | 2 (0-31) | 4 (0-31) | 5 (0-31) |  |  |
| **Perform large engine maintenance (days·month^-1^)** |  |  |  |  |  |  |
| Yes | 47 (31.5) | 22 (34.9) | 69 (32.6) | 91620 (39.4) | 0.043 | 0.631 |
| No | 102 (68.5) | 41 (65.1) | 143 (67.4) | 141284 (65.4) |  |  |
| Mean (SD) | 5.38 (10.29) | 7.33 (12.12) | 5.96 (10.88) | 7.76 (11.99) | 0.0295 | 0.436 |
| Median (Range) | 0 (0-31) | 0 (0-31) | 0 (0-31) | 0 (0-31) |  |  |
| **Construction duties (days·month^-1^)** |  |  |  |  |  |  |
| Yes | 47 (31.5) | 19 (30.2) | 66 (31.1) | 80543 (34.6) | 0.291 | 0.842 |
| No | 102 (68.5) | 44 (69.8) | 146 (68.9) | 152361 (65.4) |  |  |
| Mean (SD) | 3.78 (8.07) | 3.60 (8.01) | 3.73 (8.04) | 4.12 (8.98) | 0.284 | 0.839 |
| Median (Range) | 0 (0-31) | 0 (0-31) | 0 (0-31) | 0 (0-31) |  |  |
| **Pesticide duties (days·month^-1^)** |  |  |  |  |  |  |
| Yes | 22 (14.8) | 7 (11.1) | 29 (13.7) | 25874 (11.1) | 0.234 | 0.479 |
| No | 127 (85.2) | 56 (89.9) | 183 (86.3) | 207030 (88.9) |  |  |
| Mean (SD) | 1.54 (4.96) | 1.02 (4.41) | 1.38 (4.8) | 1.07 (4.52) | 0.208 | 0.448 |
| Median (Range) | 0 (0-31) | 0 (0-31) | 0 (0-31) | 0 (0-31) |  |  |
| **Burn Pits (days)** |  |  |  |  |  |  |
| Mean (SD) | 312.95 (267.98) | 296.32 (244.72) | 308.01 (260.82) | 331.77 (288.60) | 0.200 | 0.863 |
| Median (Range) | 253 (0-1441) | 260 (0-1282) | 255 (0-1441) | 269 (0-11170) |  |  |
| **Total Deployment (days)** |  |  |  |  |  |  |
| Mean (SD) | 412.28 (282.26) | 406.08 (264.60) | 410.43 (276.52) | 448.9 (347.23) | 0.322 | 0.926 |
| Median (Range) | 351 (2-1441) | 339 (14-1282) | 348.5 (2-1441) | 356 (0-11568) |  |  |

*^a^ Comparison of study sample to those of the AHOBP; ^b^ Comparison of PASC vs. Recovered*

### Table S4: Symptoms and Medical History

| **Characteristic, N (%)** | **PASC (N=149)** | **Recovered (N=63)** | **Study Sample (N=212)** | **AHOBPR Participants (N=232,904)** | **P-value^a^** | **P-value^b^** |
| --- | --- | --- | --- | --- | --- | --- |
| **mMRC Dyspnea Scale† (0-4)** |  |  |  |  |  |  |
| Mean (SD) | 0.95 (0.95) | 0.85 (1.07) | 0.92 (0.99) | 0.71 (0.93) | <0.001 | 0.258 |
| Median (Range) | 1 (0-4) | 0.5 (0-4) | 1 (0-4) | 0 (0-4) |  |  |
| **Difficulty Walk** |  |  |  |  | <0.001 | 0.188 |
| Yes | 118 (79.2) | 43 (68.2) | 161 (75.9) | 143450 (61.6) |  |  |
| No | 26 (17.4) | 18 (28.3) | 44 (20.8) | 82063 (35.2) |  |  |
| Other/missing | 5 (3.4) | 2 (3.2) | 7 (3.3) | 7391 (3.2) |  |  |
| **Difficulty Short walk** |  |  |  |  | <0.001 | 0.003 |
| Yes | 110 (73.8) | 32 (50.8) | 142 (67.0) | 113656 (48.8) |  |  |
| No | 34 (22.8) | 29 (46.0) | 63 (29.7) | 112027 (48.1) |  |  |
| Other/missing | 5 (3.4) | 2 (3.2) | 7 (3.3) | 7221 (3.1) |  |  |
| **Difficulty Hill** |  |  |  |  | 0.004 | 0.287 |
| Yes | 138 (92.6) | 54 (85.7) | 192 (90.6) | 192921 (82.8) |  |  |
| No | 7 (4.7) | 6 (9.5) | 13 (6.1) | 32932 (14.2) |  |  |
| Other/missing | 4 (2.7) | 3 (4.7) | 7 (3.3) | 7051 (3.0) |  |  |
| **Difficulty Stairs** |  |  |  |  | 0.001 | 0.007 |
| Yes | 129 (86.6) | 43 (68.2) | 172 (81.1) | 161804 (69.4) |  |  |
| No | 16 (10.7) | 17 (27.0) | 33 (15.6) | 64643 (27.8) |  |  |
| Other/missing | 4 (2.7) | 3 (4.8) | 7 (3.3) | 6457 (2.8) |  |  |
| **Allergies** |  |  |  |  | 0.002 | 0.175 |
| Yes | 80 (53.7) | 30 (47.6) | 110 (51.9) | 93934 (40.3) |  |  |
| No | 61 (40.9) | 25 (39.7) | 86 (40.6) | 119673 (51.4) |  |  |
| Other/missing | 8 (3.4) | 8 (12.7) | 16 (7.5) | 19297 (8.3) |  |  |
| **Asthma** |  |  |  |  | <0.001 | 0.297 |
| Yes | 42 (28.2) | 12 (19.1) | 54 (25.5) | 33927 (14.6) |  |  |
| No | 98 (65.8) | 45 (71.4) | 143 (67.4) | 182758 (78.5) |  |  |
| Other/missing | 9 (6.0) | 6 (9.5) | 15 (7.1) | 16219 (6.9) |  |  |
| **Emphysema** |  |  |  |  | 0.377 | 0.166 |
| Yes | 2 (1.3) | 2 (3.2) | 4 (1.9) | 2835 (1.2) |  |  |
| No | 138 (92.6) | 53 (84.1) | 191 (90.1) | 215470 (92.5) |  |  |
| Other/missing | 9 (6.1) | 8 (12.7) | 17 (8.0) | 14599 (6.3) |  |  |
| **Chronic bronchitis** |  |  |  |  | 0.140 | 0.159 |
| Yes | 6 (4.0) | 5 (7.9) | 11 (5.2) | 7017 (3.0) |  |  |
| No | 133 (89.3) | 50 (79.4) | 183 (86.3) | 208691 (89.6) |  |  |
| Other/missing | 10 (6.7) | 8 (12.7) | 18 (8.5) | 17196 (7.4) |  |  |
| **Chronic obstructive pulmonary disease** |  |  |  |  | <0.001 | 0.518 |
| Yes | 32 (21.5) | 11 (17.4) | 43 (20.3) | 30124 (12.9) |  |  |
| No | 101 (67.8) | 42 (66.7) | 143 (67.4) | 181434 (77.9) |  |  |
| Other/missing | 16 (10.7) | 10 (15.9) | 26 (12.3) | 21346 (9.2) |  |  |
| **Idiopathic pulmonary fibrosis** |  |  |  |  | 0.300 | 0.903 |
| Yes | 0 (0) | 0 (0) | 0 (0) | 541 (0.2) |  |  |
| No | 15 (10.1) | 6 (9.5) | 21 (9.9) | 17230 (7.4) |  |  |
| Other/missing | 134 (89.9) | 57 (90.5) | 191 (90.1) | 215133 (92.4) |  |  |
| **Future exposure-related health concern** |  |  |  |  | 0.567 | 0.294 |
| Yes | 138 (92.6) | 57 (90.5) | 195 (92.0) | 209177 (89.8) |  |  |
| No | 3 (2.0) | 0 (0) | 3 (1.4) | 3692 (1.6) |  |  |
| Other/missing | 8 (3.4) | 6 (9.5) | 14 (6.6) | 20035 (8.6) |  |  |
| **Level of concern that something breathed during deployment(s) will affect future health** |  |  |  |  | 0.610 | 0.373 |
| Yes | 138 (92.6) | 56 (88.9) | 194 (91.5) | 208431 (89.5) |  |  |
| No | 0 (0) | 0 (0) | 0 (0) | 119 (0.05) |  |  |
| Other/missing | 11 (7.4) | 7 (11.1) | 18 (8.5) | 24354 (10.5) |  |  |

*†mMRC = modified Medical Research Council dyspnea scale; ^a^ Comparison of study sample to those of the AHOBPR; ^b^ Comparison of PASC vs. Recovered*

## Dyspnea Ratings

Veterans rated their dyspnea using the modified Medical Research Council dyspnea scale at the time of AHOBPR questionnaire completion prior to COVID (Table S2), as well as post-COVID as part of this study (Table 5). Using paired statistics, we compared dyspnea reporting between these two time points separately for Veterans with PASC and those who recovered. Veterans with PASC demonstrated a mean 41% increase in dyspnea reporting (Pre vs. Post; 0.89±0.95 vs. 1.26±0.99, *p* < 0.001, Hedges’ *d* = -0.27 [-0.43, -0.11]). No increase in dyspnea was observed in those who recovered (0.81±1.06 vs. 0.67±0.84, *p* = 0.175, Hedges’ *d* = 0.12 [-0.13, 0.36]).

### Figure S1. Violin plots comparing dyspnea rating before and after COVID

**
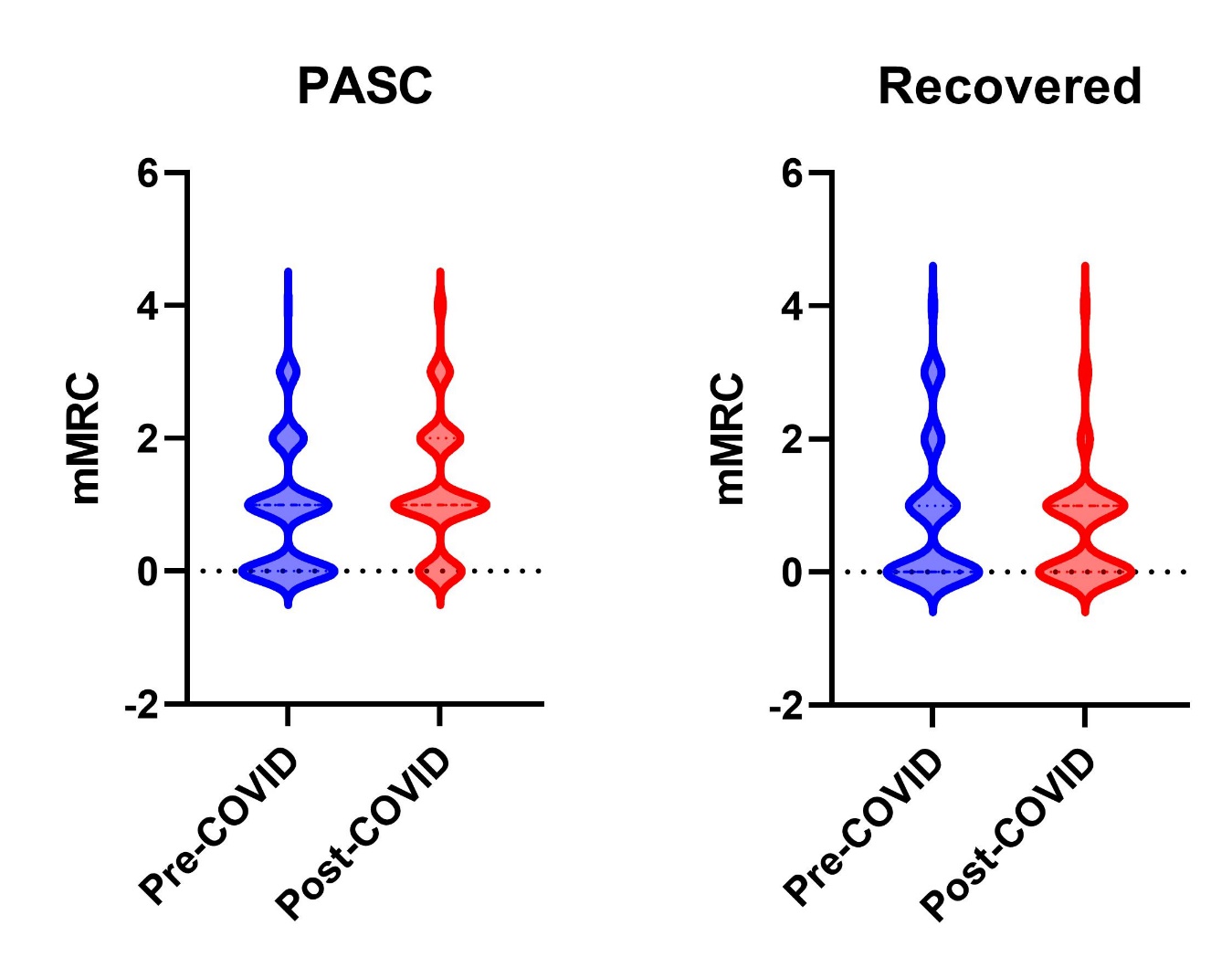
**
