# Supplementary figures and images for: Prevalence and risk factors of post-acute sequelae of SARS-CoV-2 (PASC) among veterans in the airborne hazards and open burn pit registry: a prospective, observational, nested study
Source: BMC Infect Dis. 2024 Aug 21;24:846. doi: 10.1186/s12879-024-09730-1 (PMC11337853; doi:10.1186/s12879-024-09730-1)

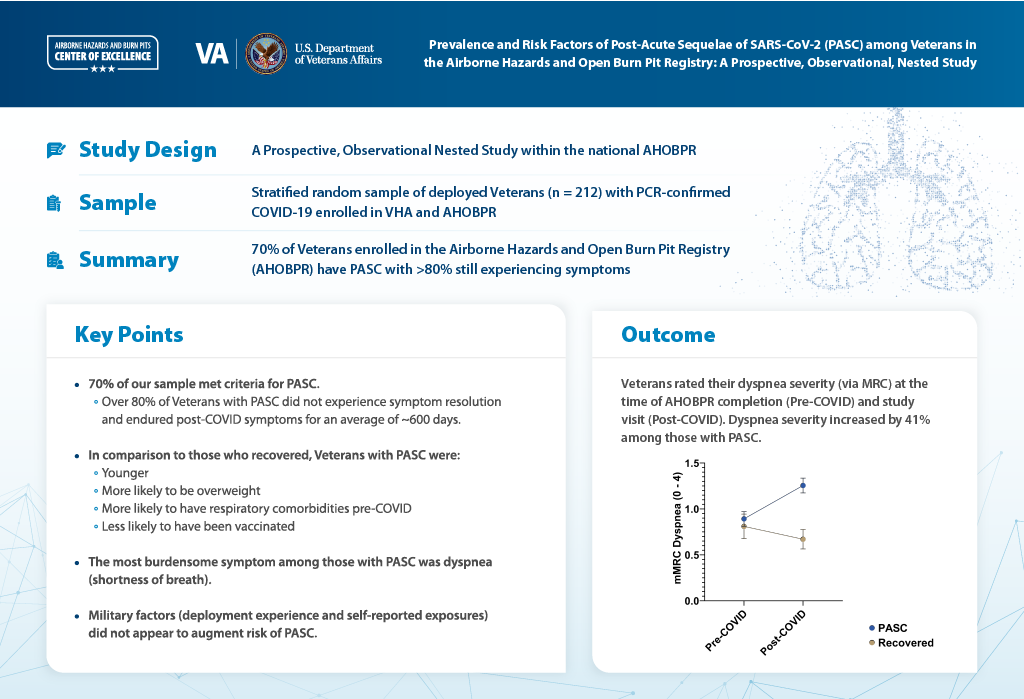

Supplement: Supplementary file 2 — Supplementary Material 2 [file 12879_2024_9730_MOESM2_ESM.png]
